# Clinical Virtual Simulation in Nursing Education: Randomized Controlled Trial

**DOI:** 10.2196/11529

**Published:** 2019-03-18

**Authors:** José Miguel Padilha, Paulo Puga Machado, Ana Ribeiro, José Ramos, Patrício Costa

**Affiliations:** 1 Nursing School of Porto; CINTESIS – Tech4edusim Porto Portugal; 2 Nursing School of Porto; CINTESIS – NursID Porto Portugal; 3 Nursing School of Porto Porto Portugal; 4 Life and Health Sciences Research Institute (ICVS), School of Medicine, University of Minho, Braga ICVS / 3B’s–PT Government Associate Laboratory, Braga / Guimarães, Portugal Faculty of Psychology and Education Sciences, University of Porto Porto Portugal

**Keywords:** clinical virtual simulation, nursing education, virtual patient, user-computer interface

## Abstract

**Background:**

In the field of health care, knowledge and clinical reasoning are key with regard to quality and confidence in decision making. The development of knowledge and clinical reasoning is influenced not only by students’ intrinsic factors but also by extrinsic factors such as satisfaction with taught content, pedagogic resources and pedagogic methods, and the nature of the objectives and challenges proposed.

Nowadays, professors play the role of learning facilitators rather than simple “lecturers” and face students as active learners who are capable of attributing individual meanings to their personal goals, challenges, and experiences to build their own knowledge over time.

Innovations in health simulation technologies have led to clinical virtual simulation. Clinical virtual simulation is the recreation of reality depicted on a computer screen and involves real people operating simulated systems. It is a type of simulation that places people in a central role through their exercising of motor control skills, decision skills, and communication skills using virtual patients in a variety of clinical settings.

Clinical virtual simulation can provide a pedagogical strategy and can act as a facilitator of knowledge retention, clinical reasoning, improved satisfaction with learning, and finally, improved self-efficacy.

However, little is known about its effectiveness with regard to satisfaction, self-efficacy, knowledge retention, and clinical reasoning.

**Objective:**

This study aimed to evaluate the effect of clinical virtual simulation with regard to knowledge retention, clinical reasoning, self-efficacy, and satisfaction with the learning experience among nursing students.

**Methods:**

A randomized controlled trial with a pretest and 2 posttests was carried out with Portuguese nursing students (N=42). The participants, split into 2 groups, had a lesson with the same objectives and timing. The experimental group (n=21) used a case-based learning approach, with clinical virtual simulator as a resource, whereas the control group (n=21) used the same case-based learning approach, with recourse to a low-fidelity simulator and a realistic environment. The classes were conducted by the usual course lecturers.

We assessed knowledge and clinical reasoning before the intervention, after the intervention, and 2 months later, with a true or false and multiple-choice knowledge test. The students’ levels of learning satisfaction and self-efficacy were assessed with a Likert scale after the intervention.

**Results:**

The experimental group made more significant improvements in knowledge after the intervention (*P*=.001; *d*=1.13) and 2 months later (*P*=.02; *d*=0.75), and it also showed higher levels of learning satisfaction (*P*<.001; *d*=1.33). We did not find statistical differences in self-efficacy perceptions (*P*=.9; *d*=0.054).

**Conclusions:**

The introduction of clinical virtual simulation in nursing education has the potential to improve knowledge retention and clinical reasoning in an initial stage and over time, and it increases the satisfaction with the learning experience among nursing students.

## Introduction

### Nursing education

The education of nursing students has always been a challenge for governments, health educators, health managers, and the students themselves to ensure the quality and safety of learning and clinical practice.

Twenty-first century students have grown up using information and communications technologies (ICT) on a day-to-day basis. The use of ICT leads to different learning processes and information structuring processes [1].

Professors and managers should bear in mind that these students are able to access information in real time, to use parallel processes and multitask; in addition, they prefer graphics to text, they function best when networked, and they need instant gratification and frequent rewards [2].

These students’ ICT skills call for innovation in the pedagogical strategies in health education underpinned by a constructivist paradigm of health education [3]. Nowadays, professors play the role of learning facilitators rather than simple “lecturers” and face students as active learners who are capable of attributing individual meanings to their personal experiences and building their own knowledge over time. An active and constructive educational environment based on challenges and learning objectives will promote deeper learning, emphasizing understanding and the application of knowledge over memorization and recall [4-8].

Innovation in simulation technologies has made available high-fidelity simulators that have supported the change in the health education paradigm. The use of high-fidelity simulators has improved the acquisition of knowledge and skills and strengthened quality and safety in clinical practice [3,9-15]. However, we have been facing challenges with the increasing cost of simulators, the difficulties of space management, and the low number of clinical scenarios available.

### Clinical Virtual Simulation

**Figure 1:**
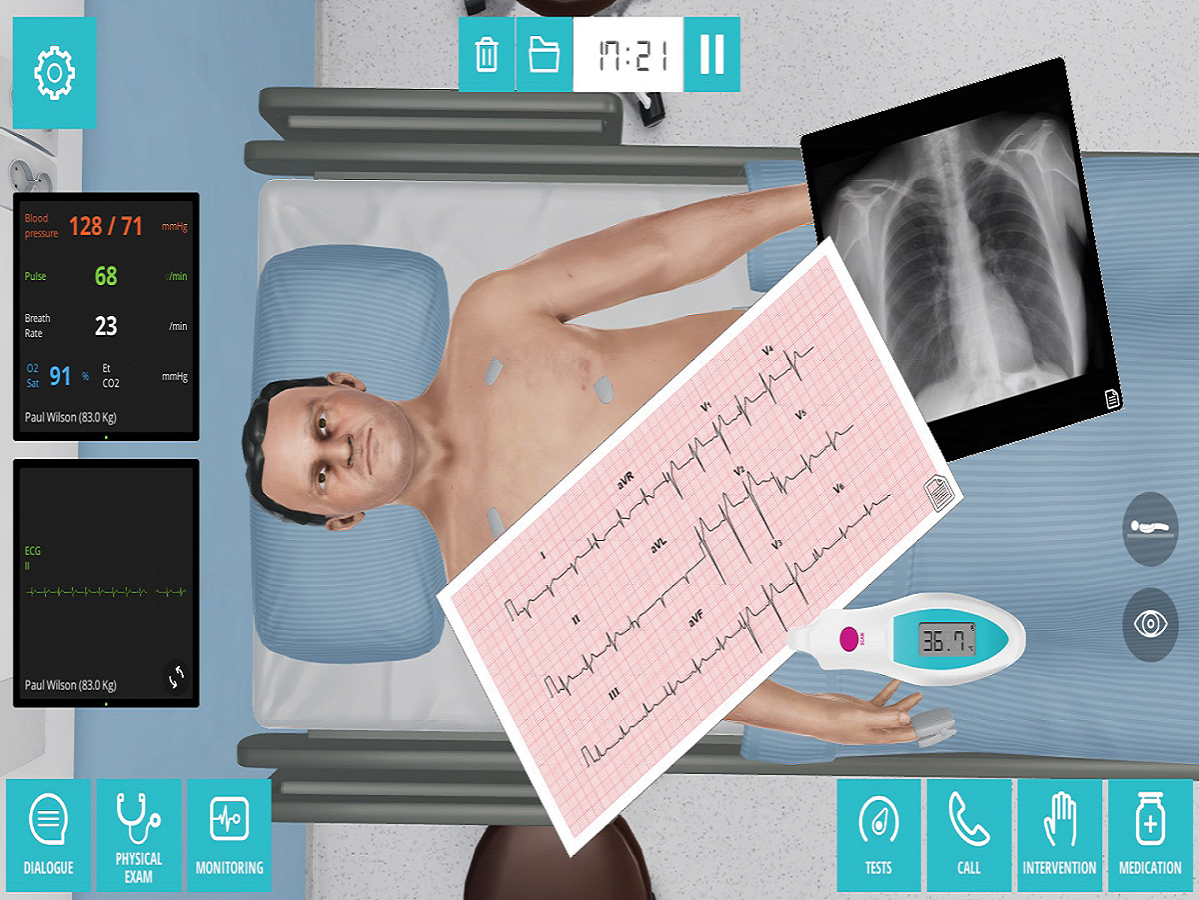
Clinical virtual simulation in hospital environment.

**Figure 2:**
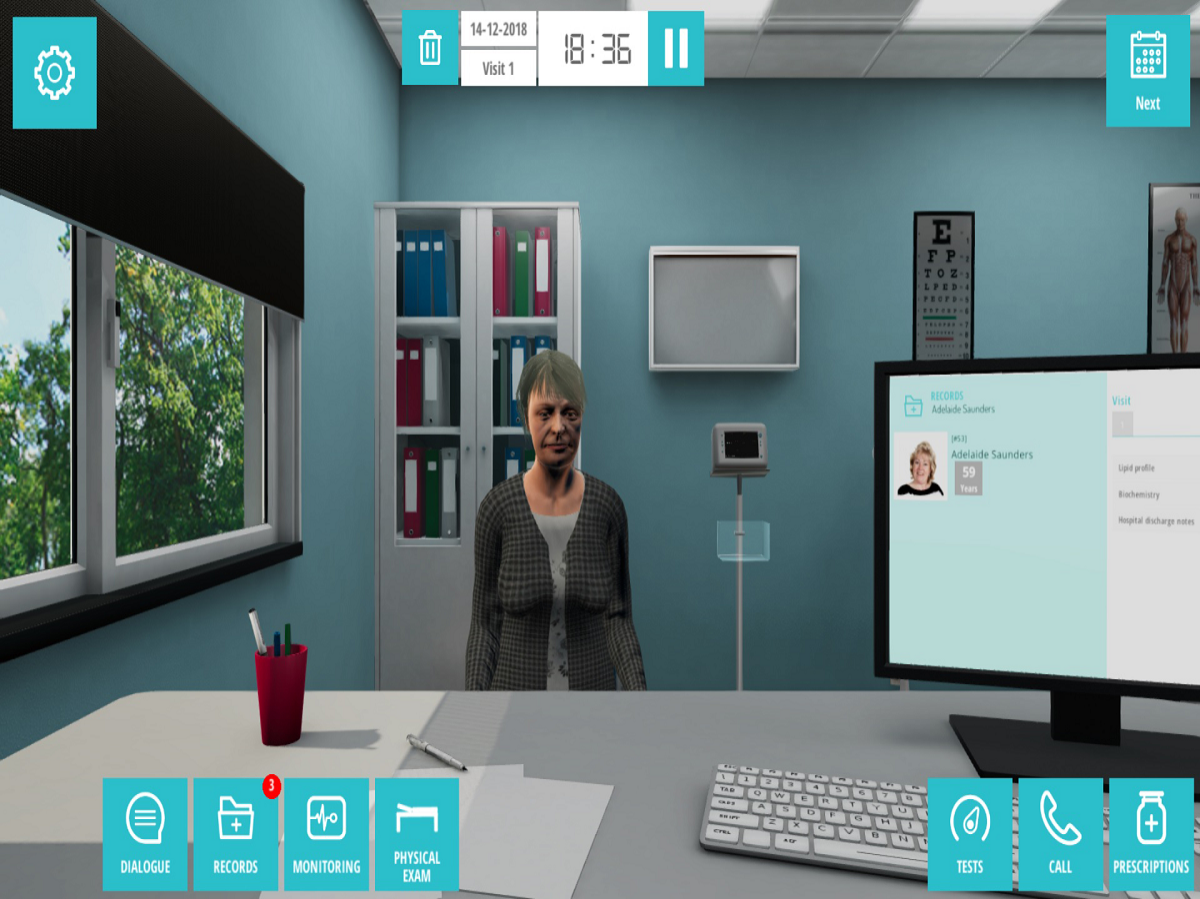
Clinical virtual simulation in environments in the community.

Developments in digital and virtual technology have eased the way to recreating reality using virtual patients [16] depicted on a computer touchscreen (clinical virtual simulation). Clinical virtual simulation is the recreation of reality depicted on a computer screen, and it involves real people operating simulated systems. It is a type of simulation that places people in a central role through the exercising of their decision-making, motor control, and communication skills [11]. Clinical virtual simulation uses virtual patients in dynamic and immersive clinical environments ranging from prehospital environments to environments in the community (Figures 1 and 2). The concept is based on the virtual patient being accessed through a variety of multimedia, screen-based interactive [17] and dynamic patient scenarios, which are supported by physiological algorithms. Clinical virtual simulation increases interaction and feedback [18] and raises both the perception of self-efficacy and the user’s satisfaction levels [19]. The use of clinical virtual simulation in the development of nursing competences improves performance [20] and competences related to psychomotor skills [21], critical thinking [22], clinical skills [23], and decision making [17].

The latest technological advances in clinical virtual simulation have improved realism and dynamic interaction, with the possibility of thousands of clinical scenarios depicted on a touchscreen table or on the Web. However, nowadays, little is known about its effectiveness with regard to students’ learning satisfaction, self-efficacy, knowledge retention, and clinical reasoning, especially when using the latest advances in clinical virtual simulation.

As professors in the field of health, we are concerned about students’ learning satisfaction and effective learning outcomes [13]. This study intended to assess the effectiveness of clinical virtual simulation in raising levels of learning satisfaction, self-efficacy, knowledge retention, and clinical reasoning among nursing students.

## Methods

A randomized controlled trial and a prospective and analytical study was conducted between March and May 2017 with a pretest and 2 posttests.

### Participants and Allocation Process

The participants were volunteer graduation students in the second year at the Nursing School of Porto in Portugal, enrolled in the course “Corporal Body Responses 1” (respiratory, cardiac, and urinary systems). This study was accomplished through an elective curriculum made available to all students. All the students enrolled in the course (N=128) were invited by email to be volunteers in the study. Those who did volunteer were invited to an initial meeting at which 56 student volunteers were present, all of whom accepted the invitation and gave informed consent. The volunteers filled out a questionnaire with sociodemographic and student data (average current course grade, number of European Credit Transfer System credits achieved as part of the nursing degree, and average grade required for admission into the degree course); these data were used in the randomization process. The anonymization of students was performed by the assignment of a number with 6 digits chosen by the student, with no possibility of the students being identified by the researchers.

The study sample size was determined considering a 1-tailed, unpaired *t* test, a type I error of 0.05, a statistical power of 0.80, and an effect size of *d*=0.80. Using G*Power3 [24] this study required a total of 42 students, 21 per group.

Students were allocated to each group through a simple random allocation using IBM SPSS Statistics version 24.

One week after the initial meeting (and after the randomization process), all the 56 volunteer students were invited to another meeting, which took place immediately before the intervention. At this second meeting, students were invited to do the first knowledge and clinical reasoning test (assessment before intervention—A0). Immediately after this, the students were directed, according to identification number (which only they were able to identify), to the classroom where they were informed about which group they had been allocated to.

Both groups received a laboratory class of 45 min, with the aim of activating knowledge and developing clinical reasoning skills in the field of the respiratory process in relation to ineffective airway clearance and hypoxia. With the experimental group, a case-based learning approach was used, with recourse to a clinical virtual simulator scenario (Body Interact) facilitated by the regular subject teacher.

The clinical virtual simulator (Body Interact) presents virtual patients backed up by a physiological algorithm that recreates a dynamic health condition that responds to user interventions. The clinical scenario is initiated by a briefing; subsequently, the user can interact with the virtual patient through dialogues, monitoring the physiological parameters, observation and physical examination, the prescription and/or analysis of complementary examinations, and the prescription of intervention and/or pharmacological treatment. The responses to and the development of the clinical case are dynamic and conditional on the decisions taken. The closure of the clinical case is determined either by the successful resolution of the scenario or by the amount of time that has elapsed (as defined by the user). Immediately after the simulation ends, a differential diagnosis interface is presented. After the simulation has concluded, the simulator provides a debriefing tool whereby 3 categories of information can be analyzed: the simulation report, the simulation timeline, and the performance report. In the simulation report, the correct differential diagnosis and the option chosen by the user are presented. All the actions carried out and the hemodynamic consequences are presented on the timeline together with all the complementary diagnosis examinations that were requested. In the simulation report, performance scores are given for 3 categories of information: physical examination, diagnosis, and therapeutic activities. In each 1 of these categories, the decisions made and their appropriacy are presented, as well as the best decision, on the basis of the evidence. The debriefing tool also provides the scientific references that support the clinical scenario and its optimal resolution.

The control group received a laboratory class of 45 min, with the same aim, using the same case-based learning approach but making use of a low-fidelity simulator and a realistic environment (pedagogical strategies that were already used in the nursing school), guided throughout by the regular subject teacher. For both groups, there was a simulation pedagogical strategy of briefing (5 min), simulation (20 min), and debriefing (20 min), with the same structure and contents.

Immediately after the end of the intervention (the laboratory class), all the students were invited to a second test (assessment after intervention—A1), and 2 months later, they were invited again to a third test (assessment follow-up—A2).

In all the knowledge assessments, we used the same true or false and multiple-choice test, which had been developed by the usual course lecturers. These knowledge assessments were based on features intrinsically related to the clinical reasoning applied within the specific scenario. In the assessment immediately after the intervention with both groups, we also assessed the students’ satisfaction levels with the simulation, and their general perception of self-efficacy.

The assessment of student satisfaction was conducted using a Portuguese version [25] of the Learner Satisfaction with Simulation Tool [19], a 10-point Likert scale. The assessment of their perception of self-efficacy was conducted with a Portuguese version [26] of the General Self-efficacy Scale [27], a 5-point Likert scale. The Cronbach alpha coefficients of the scales have been illustrated in Table 1.

### Data Analysis

We performed the Kolmogorov-Smirnov test with the Lilliefors correction to check for the normality assumption. We obtained statistically nonsignificant results for both groups in the 3 variables under study, meaning that the normality assumption was met.

The main variable under study (the development of knowledge and clinical reasoning) was obtained by the difference between the assessment before and after the intervention. Positive values reveal improvement between the 2 assessments.

To compare both groups in the relevant variables under study, we used an unpaired *t* student to compare averages.

When the homogeneity of variances assumption was violated, the Welch correction was used.

A multivariate analysis of variance (MANOVA) was performed to compare the 2 groups across the 3 measurement points.

The results were considered statistically significant for *P<*.05, and regarding effect size measures, Cohen criteria (1988) [29] were considered to rank the size of the magnitude effect (Cohen *d*: 0.2—small, 0.5—medium, and 0.8—large; partial Eta-squared: 0.02—small, 0.13—medium, and 0.26—large).

**Table 1:** Cronbach alpha coefficients for the original, for the Portuguese versions, for this study’s sample of the Learner Satisfaction with Simulation Tool, and for the General Self-efficacy Scale.

Scales	Original version, Cronbach alpha	Portuguese version		Study sample	
		Cronbach alpha	Correlation item-item total	Cronbach alpha	Correlation item-item total
Learner Satisfaction with Simulation Tool	.952	.969	.633-.823	.970	.660-.910
The General Self-Efficacy Scale (average for 25 language versions) [28]	.860	.760	.290-.530	.882	.527-.726

This study was approved by the ethics committee of the Nursing School of Porto with the number 2017/1. This randomized controlled trial does not possess a trial identifier as it is not legally required in the context of the study.

## Results

**Figure 3:**
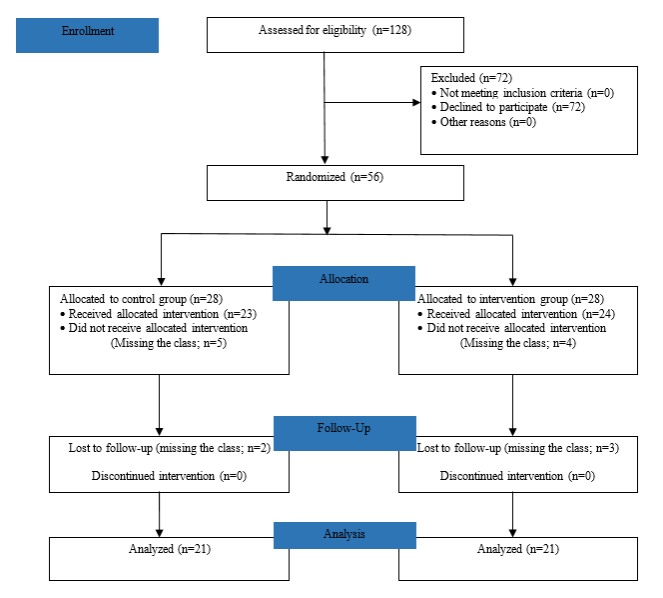
Flow diagram of sample randomization and allocation process.

A total of 42 students from the second year of a degree course participated in this study (n=21 in the experimental group and n=21 in the control group). The average age of the students was 19.9 (SD 1.99) years, and 95% (40/42) of the students were females. The flow diagram (Figure 3) represents the randomization and allocation process. Table 2 shows the results of the variables under analysis.

**Table 2:** Means of sample characteristics and study variables and SDs.

Study variables		Control group	Experimental group
**Sex, n**			
	Female	19	21
	Male	2	0
Age, mean (SD)		20.29 (2.19)	19.29 (0.46)
Mean entry grade to the degree course, mean (SD)		15.54 (1.46)	15.97 (0.85)
European Credit Transfer System credits on the degree course, mean (SD)		87.29 (6.90)	86.86 (5.41)
Degree course mean grade so far, mean (SD)		13.21 (0.67)	13.42 (0.99)
Self-efficacy perception, mean (SD)		30.14 (4.29)	30.38 (4.57)
Learning Satisfaction, mean (SD)		7.47 (1.58)	9.04 (0.55)
Knowledge assessment before intervention (A0), mean (SD)		9.87 (2.24)	10.15 (1.27)
Knowledge assessment after intervention (A1), mean (SD)		10.51 (1.89)	12.47 (1.57)
Knowledge assessment follow-up (2 months; A2), mean (SD)		10.55 (1.81)	11.93 (1.84)

### Knowledge Retention and Learning Satisfaction

The results of the students’ *t* tests showed the existence of statistically significant differences in knowledge retention after the intervention (*t*_40_=−3.656; *P*=.001; *d*=1.13), knowledge retention 2 months later (*t*_40_=−2.439; *P*=.02; *d*=0.75), and in learning satisfaction (*t*_40_=−4.309; *P*<.001; *d*=1.33). The students in the experimental group presented better outcomes in knowledge retention and learning satisfaction than students in the control group. The values of the Cohen *d* reinforce the magnitude effect of the intervention.

The MANOVA result was significant for time (Pillai Trace; *F*_2,39_=13.4, *P*<.001, partial eta squared=.407) and for the interaction term time x group (*F*_2,39_=4.45, *P*=.02, partial eta squared=.186), indicating that there are differences in the students’ levels of knowledge across time and that those differences are group dependent. Differences among moments were tested through a Bonferroni test, and significant results were observed for A0-A1 (*P*<.001), for A0-A2 (*P*=.02) but not for A1-A2 (*P*>.99; A0—assessment before intervention, A1—assessment after intervention, A2—assessment follow-up). Regarding comparisons of the different groups across time, significant differences were observed for A0-A1 (*P*<.001), for A0-A2 (*P*=.01), but not for A1-A2 (*P*=.75). No significant differences were obtained for the control group (A0-A1: *P*=.44, A0-A2: *P*=.99, A1-A2: *P*>.99).

### Self-Efficacy Perception

In self-efficacy perception, the results did not show statistical differences between the groups: *t*_40_=−0.174, *P*=.9, *d*=0.054.

Statistically significant results were also found for the overall effect of the group at the 3 measurement points: *F*_1,40_=10.2, *P*=.003, partial eta squared=.204. These results indicate that 20.4% of students’ scores across the 3 measurement points are explained by the group to which the students were assigned.

## Discussion

### Principal Findings

This paper indicates that clinical virtual simulation improves knowledge retention and initial clinical reasoning over time (2 months) and improves student satisfaction with learning, without influencing the perception of general efficiency. Clinical virtual simulation enabled a 20.4% improvement in students’ knowledge retention and clinical reasoning in the context of the study. This study showed that clinical virtual simulation is a pedagogical strategy that, combined with other strategies such as briefing, simulation, and debriefing, improves both initial knowledge retention and knowledge retention over time. Clinical virtual simulation also raises the level of satisfaction with the learning experience among nursing students. These results reveal the fit of clinical virtual simulation with the new generation’s expectations and ways of learning. The effect of the use of clinical virtual simulation as a pedagogical strategy in improving knowledge retention and clinical reasoning and students’ satisfaction levels showed a match with the features of twenty-first century nursing students. The twenty-first century nursing students had already shown high levels of usefulness, ease, and intention to use clinical virtual simulation [30]. In addition, this paper now indicates that the use of clinical virtual simulation can improve knowledge retention, clinical reasoning, and satisfaction with learning.

These results are in line with the results of other studies, where the authors found that levels of knowledge [31-33] and satisfaction [14] with the learning process improve with the use of virtual simulation.

Clinical virtual simulation brings together such strategies as gaming and problem-based learning, using an interactive and dynamic 3-dimensional technology that encourages active and critical action-based learning.

We did not find any differences in the self-efficacy perception of the students using this strategy. This is in line with the theoretical construct of Bandura’s [34] self-efficacy theory, in which the self-efficacy perception results from the interaction of different variables over time, and in this study, there was only 1 intervention with 1 class.

### Clinical Virtual Simulation in Nursing Education

Clinical virtual simulation is a complementary pedagogical strategy that provides the opportunity to improve clinical reasoning skills in students through exposure to a large number of clinical scenarios. The use of clinical virtual simulation as a pedagogical strategy should be integrated and coordinated with other pedagogical strategies in classes [35,36] and with other resources, such as high-, medium-, and low-tech simulators in use in our simulation labs to maximize the development of cognitive, affective, and psychomotor skills in the students.

This study is in line with the writings of Berman and colleagues [17]. Clinical virtual simulation is an interactive learning strategy that captures students’ intrinsic motivations and satisfaction, and it is focused on the application of foundational knowledge oriented toward a clinical learning challenge that recreates clinical scenarios with which students will be confronted in future clinical contexts. It allows a competency-based education and assessment that consequently enables a deep level of learning and the development of clinical expertise. Clinical virtual simulation can contribute toward reducing clinical error and improving the safety and quality of health care.

Clinical virtual simulation responds to the difficulties of managing laboratorial space, enabling teaching institutions to expand the number of clinical scenarios available for student training. Clinical virtual simulation makes training in the classroom context feasible and broadens the availability of scenarios in the Web environment, a feature that, in our experience, enables a tremendous increase in the number of students receiving individual training and a significant reduction in the costs of simulation use per student.

As limitations of this study, we identified the fact that it was only carried out in a single context, with second-year nursing students, and on a single course with content related only to the respiratory process. We also judge that the follow-up time was too short to fully evaluate the knowledge retention over time.

In light of these promising results, we suggest the replication of this study with a multicentric and prospective design on different health science courses.

### Conclusions

Clinical virtual simulation is a pedagogical strategy that contributes to the improvement of knowledge retention initially and over time and increases the students’ satisfaction.

This paper reveals the impact of clinical virtual simulation use in nursing education and helps professors in the field of health to be aware of its pedagogical utility and appropriacy.

These results show the potential of clinical virtual simulation to be an effective pedagogical strategy to build an educational environment that supports the development of clinical competences in the next generation of care providers, contributing toward improvements in the safety and quality of health care.

## Abbreviations

ICT: information and communications technologies
MANOVA: multivariate analysis of variance

## Appendix

Multimedia Appendix 1 CONSORT-EHEALTH checklist (V 1.6.1).
