# Correction: Clinical Virtual Simulation in Nursing Education: Randomized Controlled Trial

**DOI:** 10.2196/14155

**Published:** 2019-06-27

**Authors:** José Miguel Padilha, Paulo Puga Machado, Ana Ribeiro, José Ramos, Patrício Costa

**Affiliations:** 1 Nursing School of Porto; CINTESIS – Tech4edusim Porto Portugal; 2 Nursing School of Porto; CINTESIS – NursID Porto Portugal; 3 Nursing School of Porto Porto Portugal; 4 Life and Health Sciences Research Institute (ICVS), School of Medicine, University of Minho, Braga ICVS / 3B’s–PT Government Associate Laboratory, Braga / Guimarães, Portugal Faculty of Psychology and Education Sciences, University of Porto Porto Portugal

In “Clinical Virtual Simulation in Nursing Education: Randomized Controlled Trial’ (J Med Internet Res 2019;21(3):e11529), a paragraph from the Results section under the subheading “Self-Efficacy Perception” was erroneously duplicated in the Discussion section under the subheading “Clinical Virtual Simulation in Nursing Education”:

Statistically significant results were also found for the overall effect of the group at the 3 measurement points: *F*_1,40_=10.2, *P*=.003, partial eta squared=.204. These results indicate that 20.4% of students’ scores across the 3 measurement points are explained by the group to which the students were assigned.

The duplicated paragraph has now been removed from the Discussion.

The correction will appear in the online version of the paper on the JMIR website on June 27, 2019, together with the publication of this correction notice. Because this was made after submission to PubMed, PubMed Central, and other full-text repositories, the corrected article also has been resubmitted to those repositories.

